# Research on a Hexapod Hybrid Robot with Wheel-Legged Locomotion and Bio-Inspired Jumping for Lunar Extreme-Terrain Exploration

**DOI:** 10.3390/biomimetics11020133

**Published:** 2026-02-12

**Authors:** Liangliang Han, Enbo Li, Song Jiang, Kun Xu, Xiaotao Wang, Xilun Ding, Chongfeng Zhang

**Affiliations:** 1 School of Mechanical Engineering and Automation, Beihang University, Beijing 100083, China; 2 National Key Laboratory of Aerospace Mechanism, Shanghai 201109, China; li_en_bo@163.com (E.L.);; 3 Shanghai Institute of Aerospace System Engineering, Shanghai 201109, China; 4 College of Astronautics, Nanjing University of Aeronautics and Astronautics, Nanjing 210016, China

**Keywords:** lunar exploration, six-branched hexapod, multimodal locomotion, bio-inspired jumping

## Abstract

Exploring the lunar complex and extreme terrain presents formidable challenges for conventional lunar rovers. To address these limitations, this study proposes a novel hexapod jumping hybrid robot that incorporates a “figure-of-eight” (butterfly-shaped) six-branched wheel-legged mechanism and a jumping system that stores elastic energy via deformation of its elastic body. Inspired by the multimodal locomotion of grasshoppers, the robot dynamically switches between two operational modes: high-efficiency wheeled locomotion on relatively flat surfaces and agile jumping to traverse steep slopes and surmount large obstacles. A bio-inspired gait, inspired by the crawling patterns of a hexapod insect, is implemented using a Central Pattern Generator (CPG)-based controller to produce coordinated, rhythmic limb movements. Dynamic simulations of the jumping mechanism were conducted to optimize the critical parameters of the elastic structure and its associated control strategy. Experiments on a physical prototype were conducted to validate the robot’s wheeled mobility and jumping performance. The results demonstrate that the robot exhibits excellent adaptability to rugged terrains and obstacle-dense environments. The integration of multimodal locomotion and adaptive gait control significantly enhances the robot’s operational robustness and survivability in the harsh lunar environment, opening new possibilities for future lunar exploration missions.

## 1. Introduction

Robots serve as indispensable assets in lunar exploration, accessing regions that are inaccessible or hazardous to astronauts and conventional rovers. The lunar surface is characterized by highly complex and extreme topography—encompassing steep crater rims, boulder-strewn plains, and subsurface lunar lava tubes—posing formidable challenges to traditional mobility systems. Autonomous robots capable of navigating in such harsh environments are therefore critical for enabling in situ scientific investigations and high-fidelity data collection [1,2].

To date, most extraterrestrial rovers deployed on lunar [3] or planetary surfaces—such as China’s Yutu [4] lunar rovers and NASA’s Mars rovers [5]—employ wheeled locomotion as their primary mobility solution. The mobility mechanisms of all these planetary rovers are six-wheel rocker-bogie systems. While wheeled platforms offer advantages of high speed, substantial payload capacity, and stability on relatively flat terrain, their mobility is severely constrained in rugged or discontinuous terrain. As future lunar missions increasingly target scientifically valuable yet geologically challenging sites (e.g., polar craters and subsurface voids) [6,7], there is a pressing need for robotic systems with enhanced terrain adaptability [8].

Legged robots represent a promising alternative, owing to their superior obstacle-negotiation capabilities and adaptability to unstructured lunar terrain [9,10]. However, existing legged designs are often plagued by trade-offs between mechanical complexity, overall weight, and energy efficiency. To address this inherent limitation, recent studies have focused on hybrid locomotion strategies that integrate the strengths of multiple mobility modalities [11,12,13,14,15], such as wheel-leg hybrid locomotion, wheel-tethered hybrid locomotion, and hopping–rolling hybrid locomotion.

In response to these challenges, this study presents a six-branched wheel-legged jumping hybrid robot that integrates wheel-legged locomotion and bio-inspired jumping capabilities, inspired by grasshopper multimodal locomotion. The robot operates in two distinct, dynamically switchable modes: efficient, low-power wheel-legged locomotion on flat or gently sloped terrain and jumping mode to rapidly traverse otherwise impassable terrain when encountering large obstacles or steep gradients.

**Figure 1:**
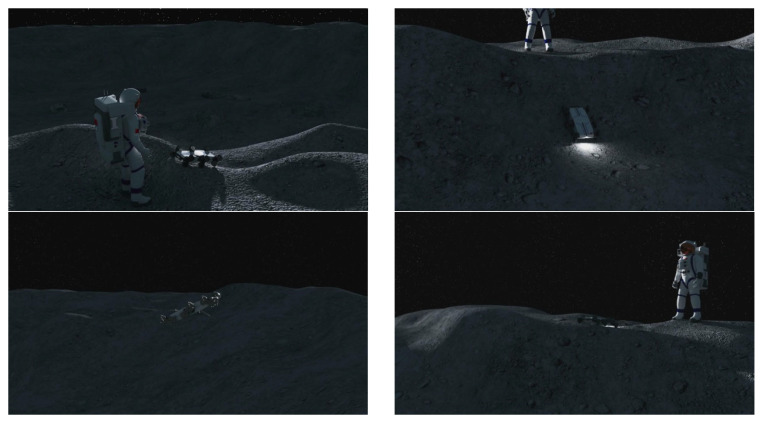
Application scenario of the wheel-legged jumping robot in manned lunar exploration missions.

By synergistically integrating the stability and robustness of hexapod locomotion with the agility and terrain passability of jumping, the proposed design significantly expands the operational envelope of lunar robotic systems. For instance, during manned lunar missions, such a robot could be deployed into an impact crater to conduct internal surveys using wheel-legged locomotion and subsequently leap out of the crater upon mission completion—enabling three-dimensional exploration of high-value lunar sites, as shown in Figure 1. This approach holds substantial promise for advancing the scope, efficiency, and resilience of future lunar scientific missions.

## 2. System Design and Implementation

### 2.1. Overall System Architecture

To meet the rigorous requirements of exploring extreme lunar terrain, the proposed six-branched hexapod wheel-legged jumping hybrid robot integrates multimodal locomotion—specifically, wheel-legged locomotion and bio-inspired jumping—with onboard scientific sensing and perception, and teleoperation capabilities.

**Figure 2:**
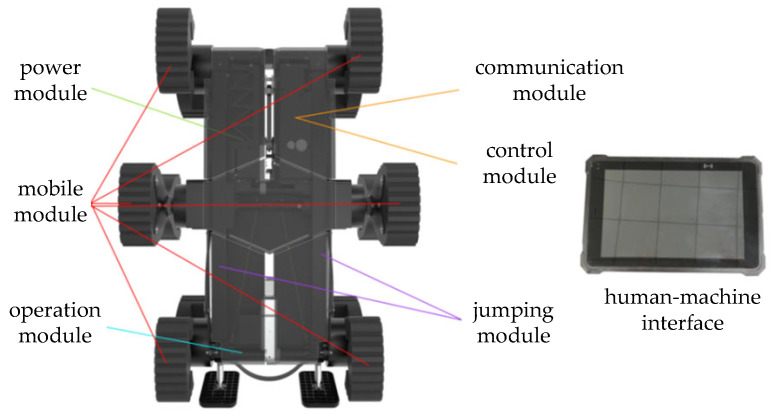
Schematic diagram of the composition of the six-branched wheel-legged jumping hybrid robot prototype.

As illustrated in Figure 2, the robot prototype consists of seven core subsystems: a mobility module (incorporating the six-branched hexapod wheel-legged mechanism), a jumping module (leveraging elastic energy storage), a central control module, a wireless communication module, a power management module, a scientific payload interface, and a human–machine interface. Such a modular architecture not only ensures functional independence among the subsystems but also facilitates coordinated operation across locomotion, perception, and command execution.

**Figure 3:**
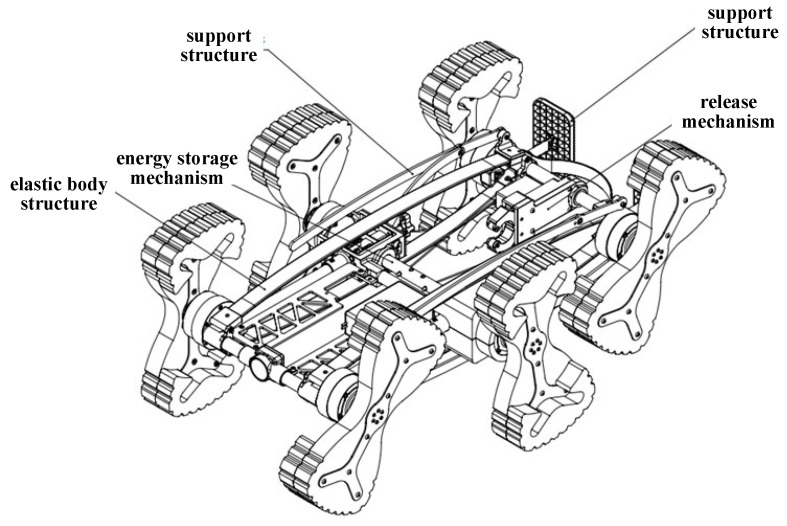
Jumping robot (without the outer shell).

**Figure 4:**
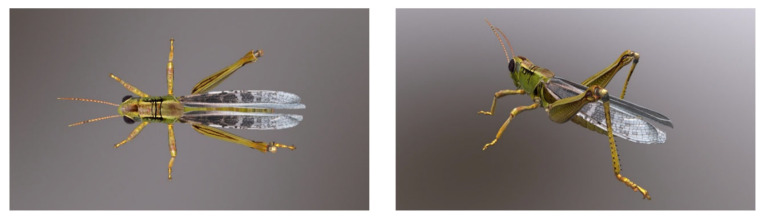
Composition of a grasshopper (hexapod structure with jumping ability in hindlegs).

From a bionic perspective [16], the six-branched hexapod robot draws direct inspiration from grasshopper locomotion. Such insects employ three primary locomotion strategies—crawling, jumping, and flying—enabling them to navigate on sandy terrain, leap over obstacles, and undertake long-distance migration. The robot emulates the crawling and jumping behaviors of grasshoppers via the following core bio-inspired mechanisms, as shown in Figure 3: (1) the six-branched wheel-legged mobility system that mimics the functional differentiation of the forelegs, midlegs, and hindlegs of the insect, as shown in Figure 4; (2) the bio-inspired jumping mechanism that emulates the forceful extension of the grasshopper hindlegs to generate rapid propulsion. This bionic design facilitates seamless transitions between stable wheeled locomotion and agile leaping, thereby enhancing adaptability in complex lunar environments.

### 2.2. Six-Branched Wheel-Legged Mobility Mechanism

To achieve stable locomotion across rugged lunar terrain, the robot leverages its core six-branched wheel-legged mobility module (Figure 2). Mirroring the functional differentiation of grasshopper legs into forelegs, midlegs, and hindlegs, the six-branched offer high reliability and kinematic redundancy, ensuring sustained mobility even if actuation failure occurs in up to two limbs [17,18]. A 3-3 gait pattern inherently ensures static stability during locomotion by maintaining three contact points with the ground at all times. Each branch adopts a simplified single-degree-of-freedom (DOF) mechanical architecture, and all six DOFs are independently driven. Coordinated gait control enables seamless transitions between wheeled locomotion and legged crawling modes, directly emulating grasshopper crawling behavior. Critically, each limb terminates in a novel, integrated dual-function wheel-leg module. Distinct from conventional C-shaped [19,20] configurations, this module features a figure-of-eight (butterfly-shaped) geometry, specifically engineered to optimize terrain interaction during both the rolling and crawling phases of the bio-inspired locomotion cycle.

**Figure 5:**
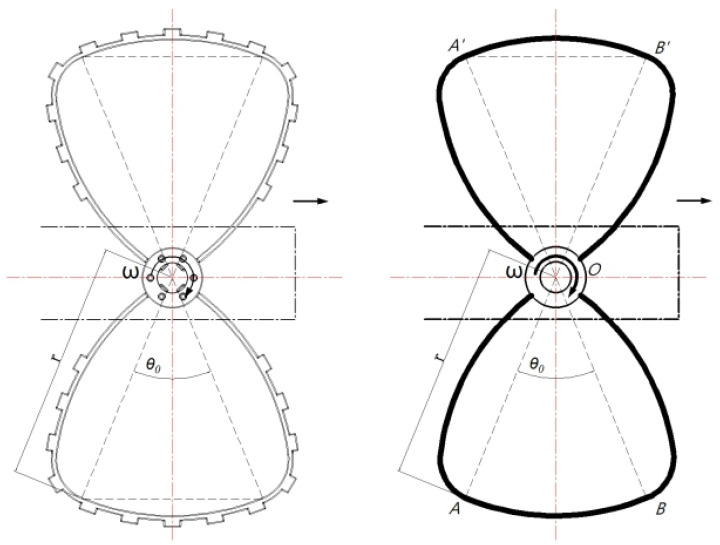
Schematic diagram of the support phase and swing of the butterfly-shaped wheel-leg.

As shown in Figure 5, during the legged crawling phase, ground contact of the butterfly-shaped wheel-leg module occurs along two arcs—$\overset{⏜}{AB}$ and $\overset{⏜}{A^{\prime }B^{\prime }}$—which are geometrically symmetric about the rotation center O. Specifically, $\overset{⏜}{AB}$ is defined at the center O with a radius r and a central angle *θ*. The drive shaft is coaxially installed at O to directly drive the module. The non-contacting segments OA and OB of the butterfly-shaped wheel-leg module do not contribute to ground support. Since the contact arcs $\overset{⏜}{AB}$ and $\overset{⏜}{A^{\prime }B^{\prime }}$ are circular arcs, the height of the center of mass (CoM) remains constant throughout the legged crawling phase, preserving a consistent vertical height r.

Let $\omega_{st}$ and $\theta_{st}$ denote the support phase angular velocity and angular displacement, respectively; $\omega_{sw}$ and $\theta_{sw}$ denote the swing phase angular velocity and angular displacement, respectively; and $\theta_{0}$ be the central angle of the arc segment $\overset{⏜}{AB}$ of the butterfly-shaped wheel-leg during the support phase; then, $\theta_{st}\;=\;\theta_{0}$ and $\theta_{sw}=\pi -\theta_{st}=\pi -\theta_{0}$.

The tripod gait employed by the six-branched hexapod robot requires three legs to maintain ground contact at all times—i.e., the duration of the support phase equals that of the swing phase; thus,

(1) $$\frac{\theta_{st}}{\omega_{st}}=\frac{\theta_{sw}}{\omega_{sw}}$$

(2) $$\theta_{0}=\frac{\pi \omega_{st}}{\omega_{sw}+\omega_{st}}$$

In the ideal scenarios neglecting slippage, when the robot moves forward, the angular velocity of the grounded butterfly-shaped wheel-leg modules corresponds to the support phase angular velocity, then, $v_{1}=\omega_{st}r$, where $v_{1}$ denotes the robot’s forward speed, and r denotes the radius of the module. The drive motor employs an integrated joint module combining the motor and gearbox, with a reduction ratio of 10:1, a rated voltage of 48 V, and an output rated speed of 391 rpm. The swing phase does not contribute to driving the robot forward; thus, the swing-phase angular velocity is maximized and set to the motor speed (391 rpm). Solving the above relationship yields

(3) $$\theta_{0}=\frac{\pi \omega_{st}}{\omega_{sw}+\omega_{st}}\geq 0.758\;rad$$

Specifically, the minimum support-phase angular displacement is 0.758 rad (43.4°), requiring that the central angle of the single-segment support-phase arc for the butterfly-shaped wheel-leg module be no less than 43.4°. The finalized structural parameters of the butterfly-shaped wheel-legged module are summarized in Table 1.

This butterfly-shaped wheel-leg module features two key technical characteristics:

(1) Under the conditions of negligible contact deformation, ground contact is confined solely to the supporting arcs. This ensures a constant center of mass (CoM) height—matching that of a pure wheeled system—thereby enhancing the stability of onboard scientific payloads during locomotion.

(2) In contrast to a conventional C-shaped wheel-leg [19,20] module with the identical contact arc length, the proposed design doubles the ground-contact duration per rotational cycle at the same motor speed, thereby enabling higher translational velocities without increasing actuation demand.

Thus, the module seamlessly integrates the efficiency of wheeled locomotion with the terrain adaptability of legged systems while mitigating the unstable motion and CoM oscillations inherent to conventional partial-wheel mechanism designs.

### 2.3. Jumping Module

This study also proposes a bio-inspired jumping mechanism that leverages the controlled elastic deformation of the robot’s compliant body. The mechanism comprises four core components: an elastic frame, an energy storage unit, a trigger-release mechanism, and a landing support assembly.

**Figure 6:**
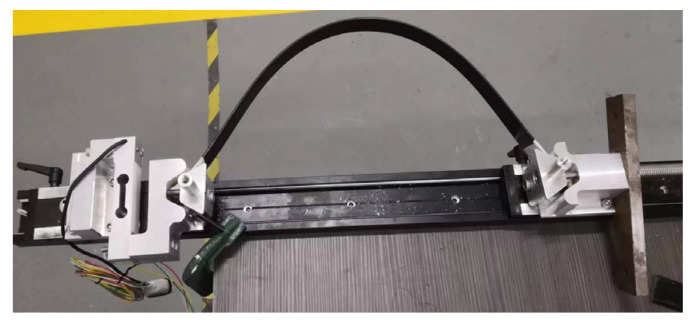
The epoxy resin material forming the elastic frame.

**Figure 7:**
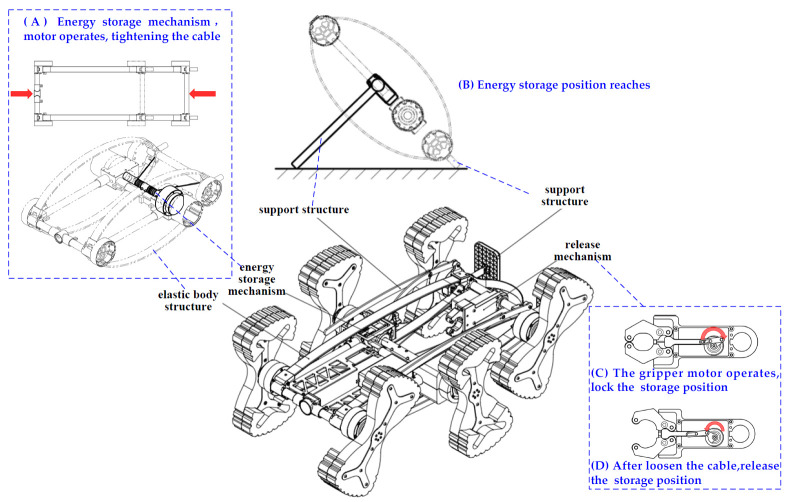
Jumping module: energy storage mechanism and release mechanism.

**Figure 8:**
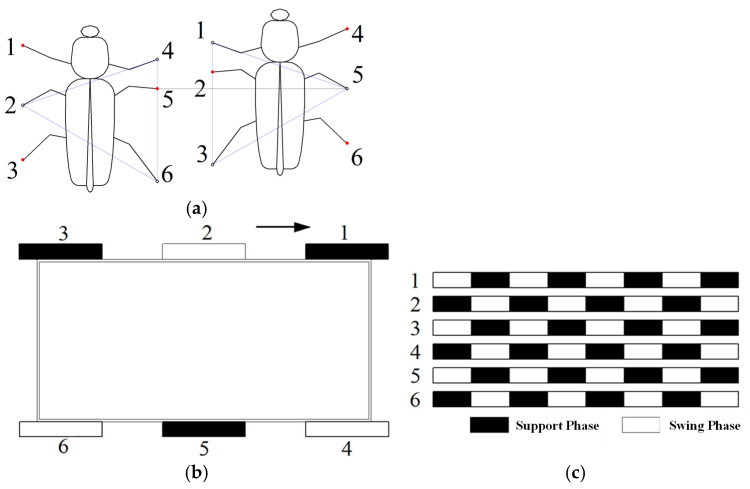
Bionic gait of robots: (**a**) Crawling process of hexapod insects. (**b**) Schematic diagram of the triangular gait. (**c**) Running diagram of the tri-angular gait.

(1) The elastic frame consists of elastic materials (Figure 6) and end-crossbeams, interconnected via hinges at the joints. Under external loading, the frame transitions from the free state to the compressed operational state, with the frame’s struts splaying outward. The energy storage unit is primarily composed of a brushless DC motor, a winch drum, and a high-strength steel cable. Mounted on the fixed frame, the motor rotates to drive the winch drum, which winds the high-strength steel cable—thereby inducing controlled elastic deformation of the elastic frame for energy storage (Figure 7).

(2) The trigger-release mechanism mainly comprises a brushless DC motor, a support frame, a mechanical gripper, a cam, a connecting rod, and a push rod, as shown in Figure 8. During operation, the motor drives the cam, which converts rotational motion into linear motion of the push rod via the connecting rod. The mechanical gripper opens when the push rod extends and closes when it retracts. The outer housing is constructed from a highly elastic composite material that not only serves an aesthetic role but also provides shock absorption and buffering to safeguard the internal components.

### 2.4. Control System

The control system adopts a proven, robust electrical and electronic architecture, with the STM32F103C8T6 serving as the main control board. This board facilitates high-speed, stable data communication with the drive motors of each leg via the RS485 communication protocol. An integrated camera captures and transmits real-time high-definition video streams to a control tablet computer over Wi-Fi.

Remote operation of the robot is achieved using a dedicated remote controller and its associated control interface. Real-time monitoring of the robot’s pitch angle is implemented to ensure operational stability. The directional control buttons govern the robot’s forward/backward translation and left/right rotation, and switch between the three discrete travel speed levels (low, medium, and high).

In jumping mode, pressing the energy storage button initiates one-touch energy storage when the system is unenergized. Upon completion of energy storage, pressing the button a second time triggers the release of stored elastic energy, propelling the robot into a jump.

Based on the aforementioned design, the robot’s achievable overall performance parameters are shown in Table 2.

## 3. Control Scheme

### 3.1. Wheeled Motion Control

#### 3.1.1. Bionic Motion Gait

Due to the unique butterfly-shaped wheel-leg structure, a single leg of the hexapod robot does not remain continuously in the support phase during rotation. The gait cycle is thus divided into two discrete phases: the support phase and the swing phase. To ensure stable, robust locomotion, a gait-planning strategy for the robot’s six legs is required.

Drawing inspiration from the crawling gaits of hexapod insects (e.g., grasshoppers), the robot’s locomotion gait is designed according to the following principle: during movement, three legs are always in the support phase—this corresponds to the triangular gait, the most widely adopted gait in hexapod insects. Specifically, one group comprises the front and hindlegs on one side, paired with the middle leg on the contralateral side, forming a triangular supporting configuration; the remaining three legs form the second group. Thus, the six legs are divided into two groups that alternately establish ground contact during forward locomotion. When one group is in the support phase, the other group is in the swing phase. As illustrated in Figure 8, Legs 1, 3, and 5 and Legs 2, 4, and 6 form two distinct groups, with a fixed inter-group phase difference of 1/2 of a cycle (corresponding to π radians in motor rotation). These two groups alternate entering the support phase to achieve straight-line locomotion.

Let $T_{st}$ denote the support time of a single leg within one gait cycle T. For the triangular gait, the duty factor β is defined as follows:

(4) $$\beta =\frac{T_{st}}{T}=0.5$$

**Figure 9:**
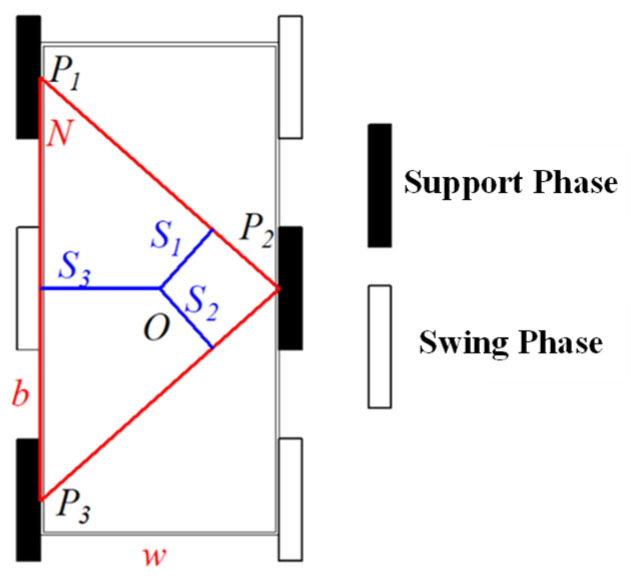
Static stability analysis.

During low-speed locomotion, the robot’s static stability is evaluated using the static stability margin (SSM) method. Specifically, the static stability margin *S* is defined as the minimum distance min{*Si*} from the projected center of mass (CoM) to each edge of the support polygon. Given the robot’s width *w* and wheelbase *b*, the static stability margin of the robot can be derived from geometric relationships as follows (Figure 9):

(5) $$S=\min\left\{\frac{wb}{2\sqrt{w^{2}+b^{2}}},\frac{w}{2}\right\}$$

Thus, in the triangular gait, the static stability margin holds true for all cases. Furthermore, the larger the robot’s width and wheelbase, the superior its static stability.

Dynamic stability is a critical criterion for evaluating the stability of fast-moving hexapod robots, and the Zero Moment Point (ZMP) approach is commonly used for dynamic stability analysis. Specifically, the ZMP is defined as the intersection of the extension line of the resultant force (encompassing the gravitational and inertial forces acting on the robot) with the support polygon N formed by the leg support points *Pi* (*i* = 1,2,…,k).

Let F represent the resultant force vector; *a* is the acceleration vector of the center of mass (CoM), and *M* is the resultant moment vector. Subscripts *x*, *y*, and *z* denote their respective components along the three coordinate axes. Additionally, *g* denotes the gravitational acceleration, *m* is the robot’s total mass, and (*x*, *y*, *z*) are the CoM coordinates of the robot in the global frame. The ZMP coordinates $\left(x_{ZMP},y_{ZMP},\;0\right)$ can be derived based on the force balance, the moment balance, and the equilibrium conditions at the *ZMP*.

(6) $$\left\{\begin{array}{c} x_{ZMP}=-\frac{M_{y}+(a_{x}z-(a_{z}-g)x)}{m(a_{z}+g)}\\ y_{ZMP}=-\frac{M_{x}+(a_{y}z-(a_{z}-g)y)}{m(a_{z}+g)} \end{array}\right.$$

**Figure 10:**
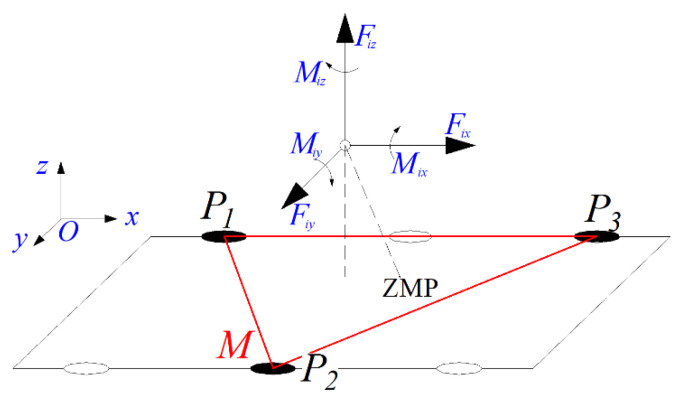
Dynamic stability analysis.

When the coordinates of this point lie within polygon N, as shown in Figure 10, it indicates that the robot is in a state of dynamic stability. Moreover, the greater the distance between the *ZMP* and the boundary, the better the dynamic stability.

#### 3.1.2. Gait Control Strategy

**Figure 11:**
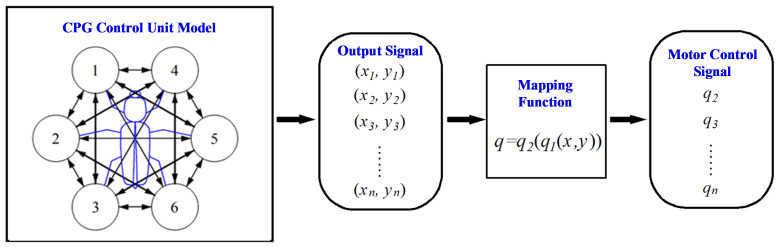
CPG control flow diagram.

For robots tasked with locomotion and exploration across unstructured, dynamically variable lunar terrain, the control strategy should be streamlined. This study employs a CPG-based control unit that requires no external sensor feedback. Mimicking the central nervous system (CNS), the CPG generates coordinated rhythmic movements and is well-suited for hexapod robots due to its strong homology with biological gaits [21,22]. CPG control signals are produced by oscillators, with common models encompassing nonlinear types such as Matsuoka, Kimura, Kuramoto, Van der Pol, and Hopf. The Hopf oscillator—featuring independent parameters and facile adjustability—was selected as the fundamental oscillator for the CPG network [23]. After mapping and subsequent processing, the CPG output signals are converted into motor joint angles to regulate the motors, as shown in Figure 11.

To ensure coordinated locomotion of the hexapod robot’s legs, a CPG network connects the corresponding Hopf oscillators to the six legs. Given that each leg of the hexapod robot in this study features only one DOF, resulting in a simplified control architecture, a strongly coupled, fully connected network is adopted, with bidirectional coupling between each pair of oscillators.

The dimensionless output signal $x_{i}$ from the CPG network control unit falls within the range of $\left[-1,1\right]$, while the rotation angle $q_{i}$ of the motor connected to the incomplete wheel spans [0,2π]. Due to the bilateral symmetry of the incomplete wheel, it exhibits a support phase and a swing phase in each of the intervals [0,π] and (π,2π]. Therefore, a mapping function must be designed to convert the CPG output signal into the motor rotation angle signal.

(7) $$q_{1}\left(x,y\right)=\left\{\begin{array}{c} -\frac{\theta_{sw}}{2}sin(\frac{\pi }{2}x)+\frac{\theta_{sw}}{2},y>0\\ \frac{\theta_{st}}{2}sin(\frac{\pi }{2}x)+\frac{\theta_{st}}{2}+\theta_{sw},y\leq 0 \end{array}\right.$$

The mapped output values obtained via this method fall within the range of 0–π, whereas the range of the motor joint angle is 0–2π. Thus, the mapped data must be further processed to double the period of the original signal. Specifically, the values remain unchanged during the original odd periods, while those in the even periods are incremented by π, i.e.,

(8) $$q_{2}\left(q_{1}\right)=\left\{\begin{array}{c} q_{1},\;\;Odd-numbered\;Cycle\\ q_{1}+\pi ,\;\;Even-numbered\;Cycle \end{array}\right.$$

**Figure 12:**
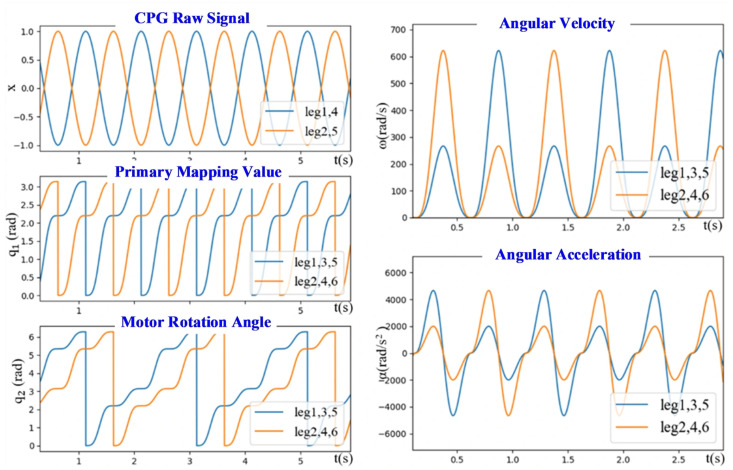
Signal processing diagram.

Therefore, the original output signals (*x*, *y*) of the CPG network are converted into motor joint angle data via the mapping function *q*_1_(*x*, *y*) and the subsequent processing *q*_2_(*q*_1_), which are then differentiated twice to derive the motor’s angular velocity and angular acceleration. As illustrated in Figure 12, the motor position command per cycle contains two support phases and two swing phases—consistent with the locomotion characteristics of the hexapod robots. Furthermore, the output motor joint angle, angular velocity, and angular acceleration exhibit the required smoothness and continuity of the locomotion.

### 3.2. Jumping Motion Control

#### 3.2.1. Jumping Solution Simulation Verification

In accordance with the development specifications, the robot has an overall mass of approximately 10 kg, with a required jumping height of over 1 m. This study presents an innovative jumping mechanism featuring an elastic outer frame structure for energy storage. To validate the feasibility of this design, multibody dynamics simulations were conducted using professional software to determine the critical design parameters, including the torque requirements of the energy storage motor, the compression stroke, and the required elastic force of the frame structure, and the maximum jumping height of the robot.

**Figure 13:**
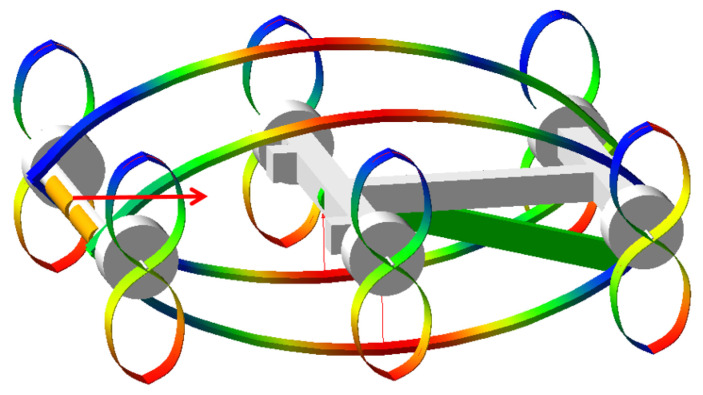
Robot rigid–flexible coupling multibody dynamics modeling.

**Figure 14:**
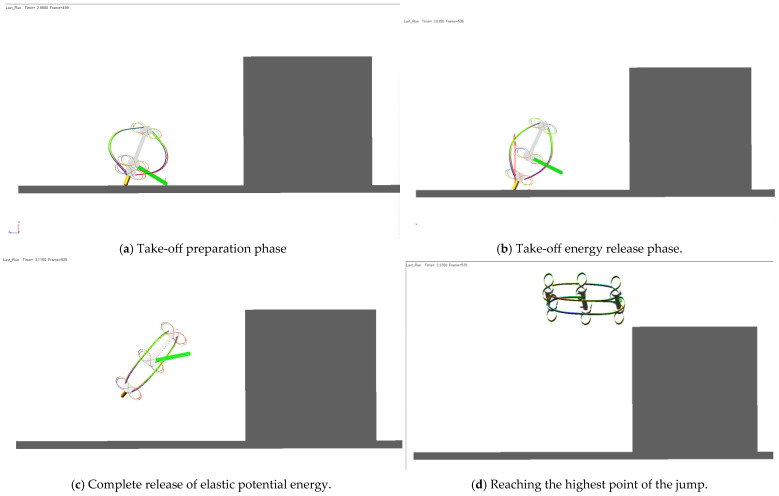
Simulation of the jumping process.

A dynamic model of the robot’s jumping motion was established, incorporating all critical moving components. The energy-storing elastic frame and elastic wheel-leg structures were modeled as flexible bodies, while all other components were treated as rigid bodies (Figure 13). Kinematic constraints between components were defined, including all relevant kinematic pairs. The most critical aspect of this analysis is the simulation of the energy storage mechanism. Specifically, a prismatic joint was defined between the rear support frame and the main control cabin support frame to simulate the relative motion between these two rigid bodies. During relative motion, the energy-storing elastic frame deforms, accumulating strain energy (Figure 14). Contact forces were defined, and simulations were performed to analyze the robot’s motion characteristics, with specific emphasis on the jumping process.

**Figure 15:**
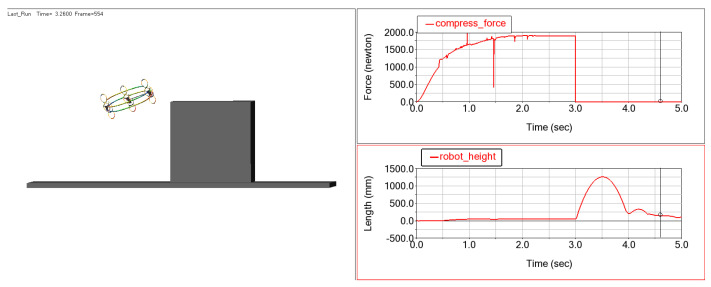
Jumping simulation results.

The key parameters of the elastic sheet—including thickness, material, and compression stroke—were optimized to ensure that the robot meets the requirement of a jumping height over 1 m. Simulation results show that when the deformation of the energy-storing frame reaches 20 cm (0.2 m), the elastic force generated is approximately 1900 N, enabling the robot to achieve a jumping height of around 1.25 m (Figure 15).

#### 3.2.2. Control Process

**Figure 16:**
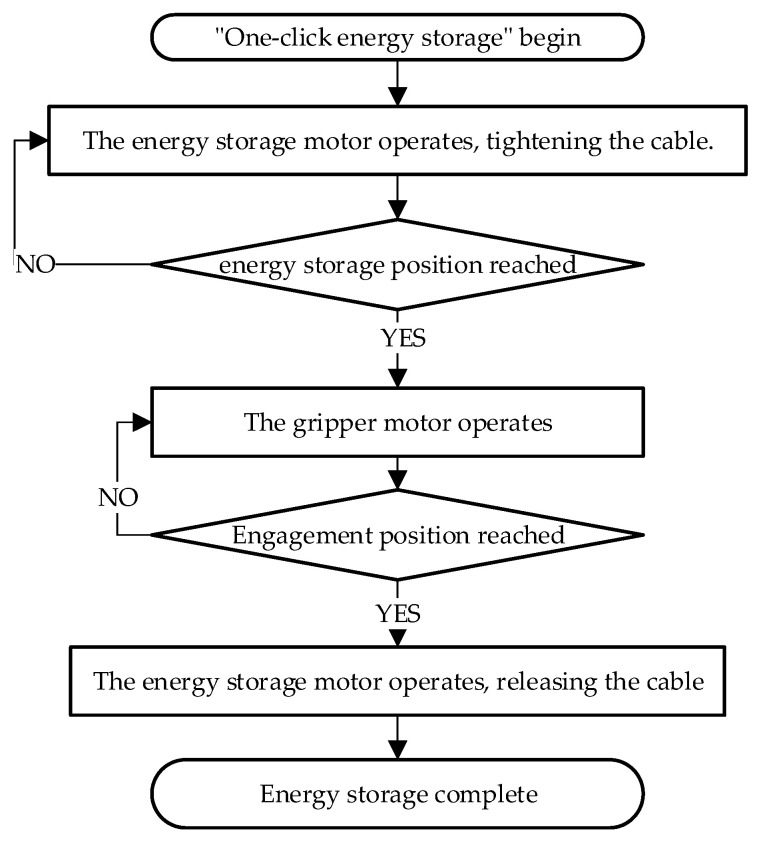
One-click energy storage control process.

The jumping motion is controlled through sequential timing, with two dedicated timing protocols—”one-click energy storage” (Figure 16) and “one-click jumping”—designed to execute the energy storage and jumping processes.

## 4. Experiments

To verify the robot’s system design and control strategy, a series of experimental validations were performed, including wheeled motion tests and jumping tests across various terrains.

### 4.1. Wheeled Motion Test

**Figure 17:**
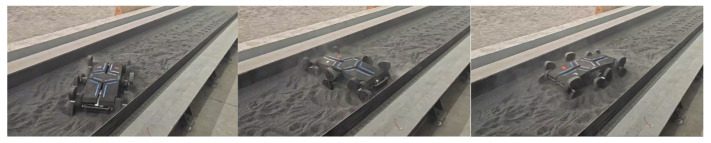
In-place turning test.

**Figure 18:**
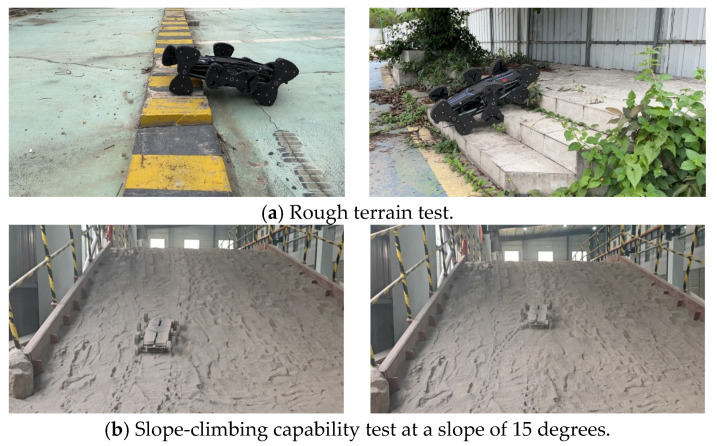
Rugged terrain locomotion capability test.

Initially, the robot prototype navigated on rough terrain via its legged gait in a simulated environment, where in-place turning tests were also conducted. The prototype operated smoothly across all motion modes, demonstrating that this mechanism configuration and locomotion gait exhibit excellent adaptability to rugged terrain. The turning radius for in-place steering is approximately 0 m, as shown in Figure 17. The slope-climbing capability on lunar regolith terrain reaches 15 degrees, as shown in Figure 18.

### 4.2. Jumping Capability Test

**Figure 19:**
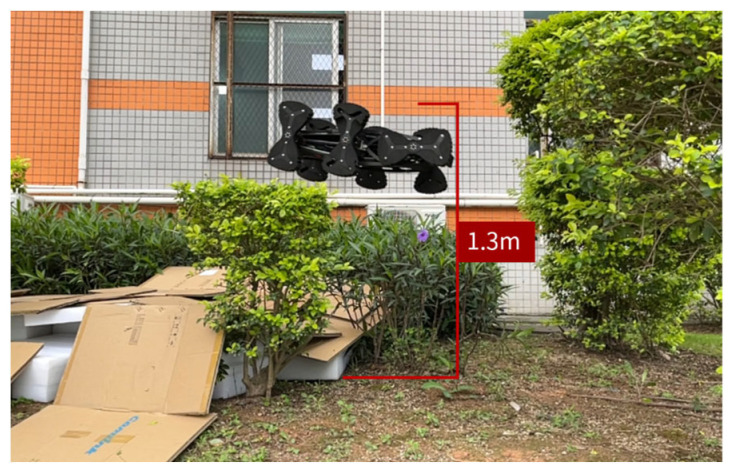
Jumping height test.

Experimental setups were established for conducting the jumping performance tests. The prototype achieved a maximum jumping height of 1.3 m (Figure 19), with both the jumping process and height meeting the design requirements. Notably, the robot’s elastic body and wheel-leg modules demonstrated excellent impact resistance during landing.

**Figure 20:**
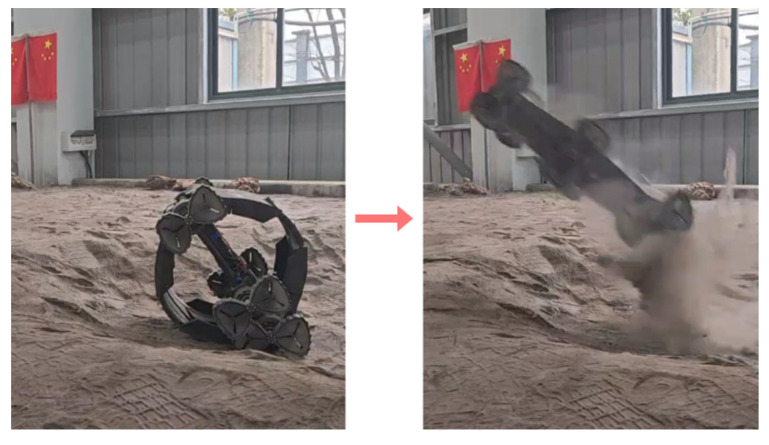
Jumping in the simulated lunar environment.

Furthermore, jumping performance tests were conducted on simulated lunar terrain (Figure 20). Test results show that the jumping height exceeds 1 m, which is slightly lower than that achieved on flat, hard ground. This phenomenon indicates that the soft lunar-like terrain attenuates the energy storage process of the elastic mechanism.

## 5. Conclusions

This study presents a novel six-branched wheel-legged jumping hybrid robot designed for lunar exploration missions. Equipped with six butterfly-shaped incomplete wheel-leg modules and an elastic body structure, the robot effectively integrates two multimodal locomotion modes: wheel-legged locomotion and jumping locomotion. Both modes were comprehensively validated through numerical simulations and physical prototype tests.

The six butterfly-shaped wheel-leg modules enable stable locomotion on rugged terrain via bio-inspired gaits, enabling smooth mobility with minimal center of mass (CoM) fluctuations. Meanwhile, the jumping locomotion mode exhibits flexible adaptability in obstacle-ridden environments. However, tests conducted on simulated lunar soil revealed that soft lunar-like terrain adversely affects elastic energy storage and impairs the instantaneous release of stored energy. These aspects represent key focus areas for design improvement in future practical applications.

In extreme lunar environments, a simple yet robust structural design, multimodal locomotion capabilities, and adaptive gaits are critical for robots to perform complex exploration tasks. This research provides a practical technical framework for future unmanned and manned lunar exploration missions, as well as for the development of next-generation lunar exploration robots.

## Figures and Tables

**Table 1:** Basic parameters of the butterfly leg structure.

Parameter	Value
Overall Dimensions/(mm)	230 × 116 × 32
Arc Leg Radius/(mm)	115
Maximum Ground Clearance/(mm)	57
Central Angle of Single-Segment Support Arc/(°)	45

**Table 2:** Parameters of the overall design.

Parameter	Value
Mass	10 kg
Overall dimensions	750 mm × 230 mm × 530 mm
Maximum wheeled locomotion speed	1.5 m/s
Minimum turning radius	0 m
Maximum jump height	1 m
Wheel-Legged locomotion actuation	6 motors
Jumping locomotion actuation	1 storage motor, 1 gripper motor

## Data Availability

The original contributions presented in this study are included in the article.
